# The Relationship between Dioxin-Like Polychlorobiphenyls and IGF-I Serum Levels in Healthy Adults: Evidence from a Cross-Sectional Study

**DOI:** 10.1371/journal.pone.0038213

**Published:** 2012-05-29

**Authors:** Octavio P. Luzardo, Luis Alberto Henríquez-Hernández, Pilar F. Valerón, Pedro C. Lara, Maira Almeida-González, Antonio Losada, Manuel Zumbado, Lluis Serra-Majem, Eva Elisa Álvarez-León, Luis D. Boada

**Affiliations:** 1 Toxicology Unit, Clinical Sciences Department, Universidad de Las Palmas de Gran Canaria, and Instituto Canario de Investigación del Cáncer, Las Palmas de Gran Canaria, Spain; 2 Radiation Oncology Department, Hospital Universitario de Gran Canaria Dr. Negrín; Clinical Sciences Department, Universidad de Las Palmas de Gran Canaria, and Instituto Canario de Investigación del Cáncer, Las Palmas de Gran Canaria, Spain; 3 Department of Biochemistry and Molecular Biology, Phisiology, Genetics and Immnunology, Universidad de Las Palmas de Gran Canaria, and Instituto Canario de Investigación del Cáncer, Las Palmas de Gran Canaria, Spain; 4 Department of Laboratory Tests, Hospital Universitario de Gran Canaria Dr. Negrín, Las Palmas de Gran Canaria, Spain; 5 Preventive Medicine and Public Health Unit, Department of Clinical Sciences, Universidad de Las Palmas de Gran Canaria, and Instituto Canario de Investigación del Cáncer, Las Palmas de Gran Canaria, Spain; 6 Preventive Medicine Service, Complejo Hospitalario Materno Insular de Gran Canaria, and Instituto Canario de Investigación del Cáncer, Las Palmas de Gran Canaria, Spain; California Pacific Medicial Center Research Institute, United States of America

## Abstract

**Objective:**

Insulin-like growth factor I (IGF-I) and dioxin-like polychlorobiphenyls (DL-PCBs) have been associated with the pathogenesis of several diseases like cancer, diabetes and growth disorders. Because it has been suggested that organohalogenated contaminants could influence IGF-I levels in adults, the potential relationship between DL-PCBs and IGF-I serum levels was studied in 456 healthy adults from a representative sample of the general population of the Canary Islands (Spain).

**Design:**

Free circulating serum levels of IGF-I and IGFBP-3 were measured through an ELISA methodology, while the serum levels of the 12 DL-PCBs congeners (IUPAC numbers # 77, 81, 105, 114, 118, 123, 126, 156, 157, 167, 169, and 189) were measured by gas chromatography/mass spectrometry (GC-MS).

**Results:**

DL-PCBs 156 and 167, Total DL-PCBs body burden (∑PCBs: sum over the 12 measured DL-PCBs), and Total toxic burden (in terms of toxic equivalence to dioxins: ∑TEQs) showed a trend of inverse association with IGF-I serum levels in the whole studied population. After adjusting for potential confounders, including gender, body mass index (BMI), age, and IGF-binding protein-3 (IGFBP-3), younger (18–45 years) women with lower BMI (<27 kg/m^2^) and detectable levels of DL-PCB-156 showed significantly lower IGF-I levels than those in the same age and BMI subgroup with non-detectable levels of DL-PCB-156 (*p*<0.001). Similarly, ∑PCBs and ∑TEQs showed a tendency to an inverse association with IGF-I levels in the same group of women (*p*=0.017 and *p*=0.019 respectively).

**Conclusions:**

These findings suggest that DL-PCBs could be involved in the regulation of the IGF-system in a way possibly influenced by gender, age and BMI. Although these results should be interpreted with caution, such circumstances could contribute to explain the development of diseases associated to the IGF system.

## Introduction

Insulin-like growth factor-I (IGF-I) is an amino acid peptide synthesized in the liver in response to growth hormone (GH). IGF-I may circulate in one of two states, either free or bound to one of six different binding proteins, with >90% bound to the IGF binding protein 3 (IGFBP-3) [1]. The regulatory role of IGF-I on cellular differentiation and proliferation, as well as on a number of tissue-specific functions, is well known [2]. In addition, IGF-I has been found to be involved in the development of a number of diseases, such as diabetes, cancer, and growth disorders [3], [4], [5].

Serum levels of IGF-I are influenced by gender, age, body mass index (BMI), and dietary and lifestyle factors [6]. IGF-I levels show sexual dimorphism, with higher levels in men than in women, especially in older people [7], although the influence of gender is not completely understood [8]. An evident inverse relationship between age and IGF-I levels has been described in adults [9], [10]. An inverse association between IGF-I and BMI has also been reported [6], [11] and recently published studies indicate that such association is non-linear [12]. Dietary factors may play a role on IGF-I levels [13]. It was recently proposed that chemical pollutants in food and environment (i.e. organochlorine pollutants) should also be taken into account [14]. Exposure to environmental pollutants, such as dioxins and polychlorobiphenyls (PCBs), may affect IGF-I homeostasis [15], [16] although not yet demonstrated such associations in humans.

PCBs are environmentally and biologically persistent polyhalogenated aromatic hydrocarbons [17]. PCBs are mixtures of up to 209 different congeners, although only 36 of them are environmentally relevant [18]. Since they are lipid-soluble and resistant to chemical and biological degradation, PCBs intake leads to life-long bioaccumulation, both in animals and humans, and to biomagnification through the food chain [17]. Due to their persistence both in the environment and living organisms, these substances were banned in 1970s and 1980s in most Western countries [19].

PCBs have been detected in biological samples from populations all over the world [20], [21], [22]. A number of factors seem to influence PCBs serum levels in humans (e.g. gender, age, BMI and diet or lifestyle factors). In an earlier study [23], we reported the overall PCBs serum levels in a representative sample of the same population involved in this study, namely adults living in the Spanish archipelago of the Canary Islands. The serum levels of DL-PCBs residues in this Canarian population were lower than those reported for other Western populations [23].

Exposure to PCBs has been associated with several health outcomes, especially when the so-called dioxin-like PCBs (DL-PCBs) are involved – 12 PCB congeners with toxic effects similar to those of dioxins [24]. Since 1987, PCBs have been considered as probable human carcinogens (Group 2A) by the International Agency for Research on Cancer (IARC) [25]. Moreover, evidences show that exposure to these chemicals may be associated with alterations in memory, learning, and other neuropsychological effects [26], diabetes [27], [28], and restricted growth in children [29]. It was recently suggested that these chemicals could also induce disturbances in growing hormone axis activity and growth rate [30].

The aim of this study was to explore the association between DL-PCBs and IGF-I serum levels in healthy subjects from a population previously studied about the levels of contamination by PCBs. [23].

## Materials and Methods

### Subjects group and collection of blood-samples

This study included 456 adult subjects (203 men and 253 women) aged 18 to 73 years, who were enrolled during the Canary Islands Nutritional Survey (ENCA). The ENCA nutritional survey was conducted in 1997–1998 in the seven major Islands of the Canary Archipelago (Spain). The sample was stratified in two stages, and it was representative of the population between 6 and 75 years of age for both genders. Participants were randomly selected from the official census. A total of 1747 healthy subjects participated in the first part of the study. This part consisted in two individual interviews with questions about dietary variables, life habits, and health condition. The participants had blood samples extracted after 12-h fasting in order to determine biochemical parameters and the presence of DL-PCBs. A total of 783 subjects participated in the biochemical part (participation rate, 44.8%). Data about DL-PCB residues in the adult subgroup of subjects were available in 456 people [31]. All subjects received and signed a written informed consent prior to blood extraction. Only subjects who signed the informed consent were included. The study was approved by the Research and Ethics Committee of the Complejo Hospitalario Universitario Insular-Materno Infantil (Las Palmas de Gran Canaria, Spain). The participants were not compensated for their collaboration in any way. Table 1 shows the characteristics of the population under study.

**Table 1:** Biological characteristics of the studied population, N (%).

Characteristic	Total	Male	Female
	456 (100)	203 (44.5)	253 (55.5)
**Age** (years)			
*18–34*	107 (17.6)	47 (23.2)	60 (23.7)
*35–50*	139 (22.9)	57 (28.1)	82 (32.4)
*50–65*	141 (23.2)	64 (31.5)	77 (30.4)
*>65*	69 (11.4)	35 (17.2)	34 (13.5)
**BMI** (kg/m^2^)			
*<18.5*	10 (2.3)	3 (1.5)	7 (2.9)
*18.5–24.99*	164 (35.9)	72 (35.4)	92 (36.4)
*25–29.99*	175 (38.4)	79 (39.0)	96 (38.0)
*>30*	107 (23.4)	49 (24.1)	58 (22.7)

### Determination of IGF-I and IGFBP-3

Free circulating serum levels of IGF-I and IGFBP-3 were measured the subgroup of people with data about serum levels of DL-PCBs (456 adult people), in the Department of Laboratory Tests from the Hospital Universitario de Gran Canaria Dr. Negrín (Canary Islands, Spain) using a commercially available enzyme linked immune sorbent assay (ELISA) kit (Diagnostic Systems Laboratories, Webster, TX, USA); a useful assay widely used with large sample sizes [9], [14], [32], [33]. All the samples were analyzed in duplicate. Data of IGF-I serum levels were available for all the study population. The overall intra-batch coefficients of variation were lower than 10% for both proteins. The average inter-batch coefficients of variation were 9% for IGF-I and 12% for IGFBP-3. The coefficients of variation detected coincided with those reported by the manufacturer.

### Analytical procedure: sample preparation and laboratory tests

The sample preparation and laboratory tests procedures are described elsewhere [23]. Briefly, serum aliquots (2–3 ml) were subjected to solid-phase extraction and subsequent gas chromatography/mass spectrometry (GC/MS) with appropriate internal standards. The analytes in this study were the DL-PCBs congeners with IUPAC numbers # 77, 81, 105, 114, 118, 123, 126, 156, 157, 167, 169, and 189. Chromatography was performed with Thermo-Finnigan TRACE DSQ GC/MS equipment. Standards and samples were injected (2 ml) in the splitless mode. Tetrachloro-m-xylene was used as a surrogate and PCB 202 as the internal standard. The limit of quantification (LOQ) for DL-PCB congener 118 was 0.010 ng/ml; while the LOQ for the rest of analytes was 0.001 ng/ml. The standard analytes for this study were purchased from Dr. Ehrenstorfer (Riedel-de Hagen, Sigma-Aldrich Laborchemikalien GmbH, Germany).

Serum DL-PCBs concentration values measured by chromatography were lipid-adjusted (lipid-adjusted serum concentrations of lipophilic compounds provide better estimations of the burden) [34]. Total cholesterol and triglycerides were measured with an automatic Hitachi Analyzer 717 (Boehringer Manheim, IN, USA). Total serum lipids were estimated by using the formula established by Phillips [35].

Throughout this work, Total DL-PCBs body burden (∑DL-PCBs) expresses the sum over the measured serum levels of the 12 DL-PCBs; potential toxicity (in terms of toxic equivalent to dioxins: TEQs) was estimated using the toxicity equivalent factors (TEF) reviewed by the World Health Organization (WHO) in 2005 [36]; and Total TEQs (∑TEQs) expresses the sum over the TEQs corresponding to the measured serum DL-PCBs.

### Statistical analysis

Database management and statistical analysis were performed with the PASW Statistics v 17.0 statistical software (SPSS Inc., Chicago, IL, USA). Since the serum levels of PCBs, IGF-I and IGFBP-3 did not follow normal distributions, results were expressed as median and 5^th^ and 95^th^ percentiles. Between-group differences in PCB levels were analyzed with the non-parametric Mann–Whitney U-test and the Kruskal Wallis test. Correlation between PCB levels and continuous variables was analyzed with the Spearman's correlation coefficient. Multivariate analysis was performed using the variance component analysis stratifying the population according to gender, age (above or below the mean: 46 years for women, and 47 years for men) and BMI (above or below the mean: 27 kg/m^2^ for both), and after adjusting by IGFBP-3, and gender or age or BMI [9], [32], [37], [38], [39]. Because of the large number of serum samples which present DL-PCBs serum values below the LOQ, samples were considered to be either detectable (D-sample) or non-detectable (ND-sample) for every congener and were introduced into the model as categorical factor. However, because of the large number of samples with ∑DL-PCBs and ∑TEQs above the LOQ, these variables were categorized into gender-age-BMI-specific tertiles (lowest ∑DL-PCBs and ∑TEQs values in the first tertile, intermediate values in the second tertile, and highest values in the third tertile) and introduced into the model as categorical factors. *P* values<0.05 (two-tails) were considered to be statistically significant.

## Results

The studied population included healthy adults with similar average age and BMI values for men and women (47.7 years and 26.9 kg/m^2^ for men; 46.4 years and 26.9 kg/m^2^ for women). Table 2 shows that the median free circulating IGF-I values did not differ significantly between genders, while evidencing the physiological profile of serum IGF-I levels, which significantly decrease with increasing age (*p*<0.001 for both genders; data not shown) and with increasing BMI (*p*=0.004 for men and *p*<0.001 for women; data not shown). Free circulating serum levels of IGFBP-3 were significantly different according to gender and age (Table 2).

**Table 2:** Distribution of IGF-I and IGFBP-3 (ng/ml) in the studied population.

Characteristic	IGF-I[median (p5-p95)]	IGFBP-3[median (p5-p95)]
**Whole series**	157.0 (73.0–277.5)	3998.0 (2607.0–5775.0)
**Gender**		
Male	159.0 (87.0–278.0)	3938.0 (2427.8–5853.6)
Female	155.5 (70.9–273.4)	4044.0 (2835.2–5770.8)
*P*	0.428	0.025
**Age** (years)		
18–34	201.0 (125.2–320.8)	4205.5 (3094.4–6067.4)
35–50	170.0 (101.6–246.4)	4074.0 (2918.3–5912.0)
50–65	145.0 (64.3–237.5)	3763.0 (2318.5–5528.7)
>65	133.0 (39.0–227.0)	3588.0 (2286.9–5260.5)
*P*	<0.001	<0.001
**BMI** (kg/m^2^)		
<18.5	210.0 (113.0–307.0)	4814.5 (3110.0–5410.0)
18.5–24.99	178.0 (100.4–304.8)	3939.0 (2627.3–5825.1)
25–29.99	151.0 (83.4–263.3)	3998.0 (2563.8–5543.6)
>30	140.0 (57.7–243.6)	3978.0 (2470.8–5952.0)
*P*	<0.001	0.135

*Abbreviations:* p5 represents the 5th percentile and p95 represents the 95th percentile.

*P* values correspond to comparison between characteristics, for IGF-I and IGFBP-3 (Kruskal-Wallis test).

Subjects with non-detectable levels of DL-PCBs were younger than those showing detectable levels of DL-PCBs (*p*<0.0001, 42.2 vs. 50.1 years old, respectively). As a consequence, subjects showing undetectable DL-PCBs serum levels had higher IGF-I median values than those showing detectable DL-PCBs serum levels (*p*=0.003, 173.0 vs. 153 ng/ml, respectively).

A positive correlation between age and DL-PCBs was evident (R=0.327, *p*<0.0001). This association was also observed indistinctly among men (R=0.374, *p*<0.0001) and women (R=0.274, *p*<0.0001). However, we did not find any association related to BMI.

Three out of the total 12 measured DL-PCB congeners (# 77, 105 and 114) were not found in any of the samples, while congeners 156 and 167 were detected in more than 40% of samples, with percentile 95^th^ concentrations as high as 10.9 and 8.7 ng/g lipid, respectively (Table 3). However, the most relevant congeners in terms of high TEF values, PCBs 126 and 169, were detected in a low percentage of samples (0.7 and 6.1%, respectively). As previously described [23], the median TEQ value corresponding to the measured ∑DL-PCBs was low (0.1 pg/g lipid). However, it should be highlighted that TEQs levels reached values of 40.2 pg/g lipid in serum samples included in percentile 95^th^ (Table 3).

**Table 3:** Distribution of DL-PCBs concentrations (ng/g lipid) and TEQs (pg/g lipid) in adult blood serum in the population of the Canary Islands.

Congener	Concentration median (p5–p95)	TEQs median(p5–p95)	(%)
**DL-PCB (Non-ortho)**			
*PCB-77*	<LOQ	N.A.	0.0
*PCB-81*	0.0 (0.0–0.0)	0.0 (0.0–0.0)	4.6
*PCB-126*	0.0 (0.0–0.0)	0.0 (0.0–0.0)	0.7
*PCB-169*	0.0 (0.0–1.3)	0.0 (0.0–38.9)	6.1
**DL-PCB (Mono-ortho)**			
*PCB-105*	<LOQ	N.A.	0.0
*PCB-114*	<LOQ	N.A.	0.0
*PCB-118*	0.0 (0.0–23.1)	0.1 (0.0–0.7)	18.9
*PCB-123*	0.0 (0.0–0.0)	0.0 (0.0–0.0)	0.9
*PCB-156*	0.0 (0.0–10.9)	0.0 (0.0–0.3)	48.5
*PCB-157*	0.0 (0.0–0.0)	0.0 (0.0–0.0)	0.2
*PCB-167*	0.0 (0.0–8.7)	0.0 (0.0–0.3)	43.9
*PCB-189*	0.0 (0.0–0.0)	0.0 (0.0–0.0)	1.5
**ΣDL-PCBs**	2.8 (0.0–56.8)		60.3
**ΣTEQs**	0.0 (0.0–0.4)	0.1 (0.0–40.2)	60.9

*Abbreviations:* LOQ, limit of quantification; N.A., not applicable. p5 and p95 represent the 5^th^ and 95^th^ percentiles respectively. ∑DL-PCB: sum over the dioxin-like PCB levels (IUPAC numbers 77, 81, 105, 114, 118, 123, 126, 156, 157, 167, 169, and 189). ∑TEQs: sum over TEQs for the DL-PCBs.

**Figure 1:**
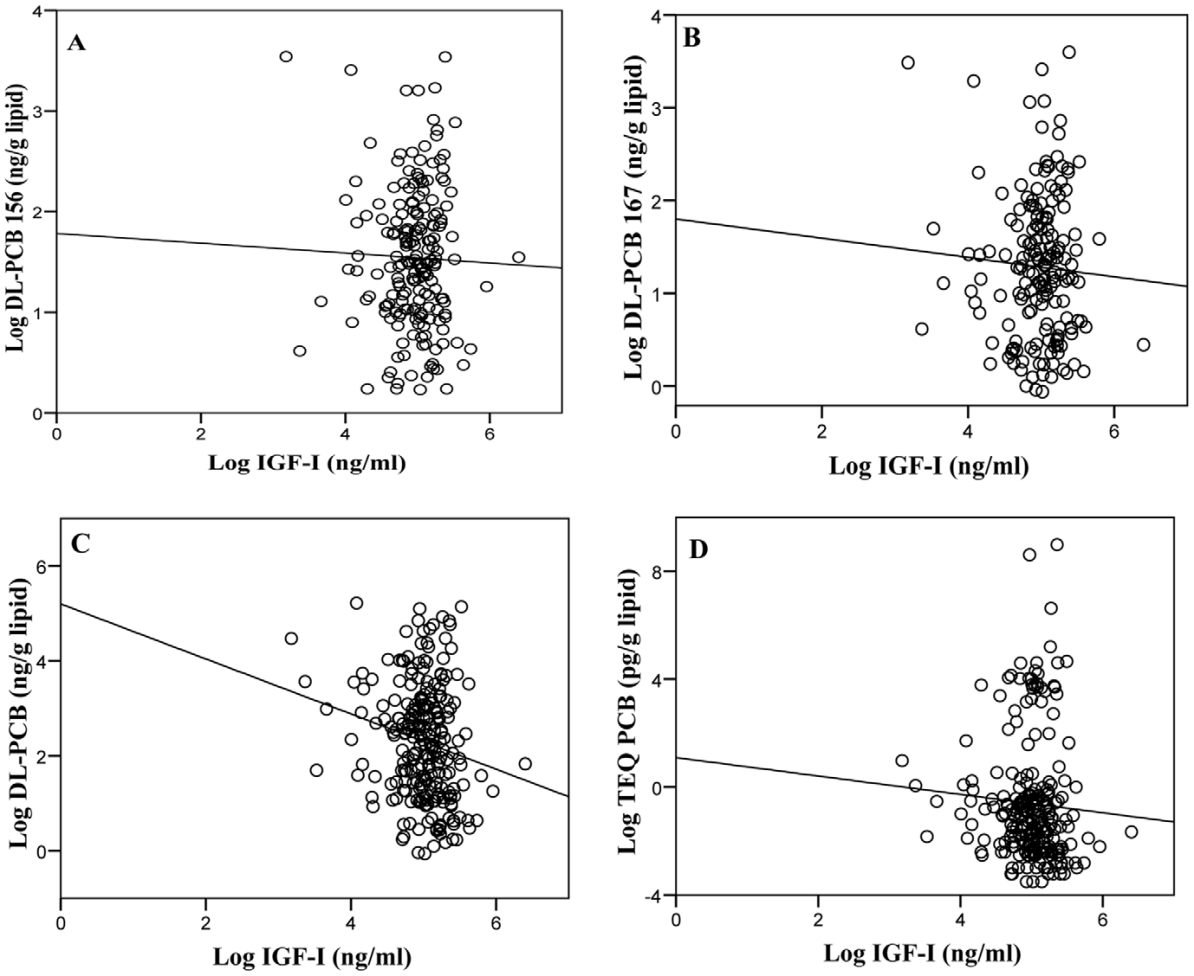
Scatter plot of log IGF-I serum levels and log of DL-PCB 156 (A), PCB 167 (B), ∑DL-PCBs (C), and ∑TEQs serum levels (D).

The serum levels of IGF-I and DL-PCBs showed a trend of inverse association in the whole population. Thus, serum IGF-I values decreased with increasing PCB-156, PCB-167, ∑DL-PCBs and ∑TEQs (R=−0.210, *p*<0.001; R=−0.140, *p*=0.006; R=−0.196, *p*<0.001; and R=−0.184 *p*=0.001, respectively). The relationship between IGF-I and the above variables was evident in a neperian log-transformed scatter plot (Figures 1A, 1B, 1C and 1D). This trend was also evident in a separate analysis of data from men (*p*<0.05 for all comparisons) and women (*p*<0.01 for all comparisons) (not shown).

Since gender, age, BMI and IGFBP-3 play an important role in IGF-I serum levels, and gender, age and BMI also seem to be important determinants of DL-PCBs distribution [23], we carried out a multivariate analysis (stratifying the population sample by gender, age and BMI, and adjusting for IGFBP-3 and age, gender or BMI) in order to evaluate the potential role of DL-PCBs on IGF-I serum levels. Results of this analysis showed the same negative-relationship tendency described above both for men and women, although some associations failed to reach significance (possibly due to the small size of some subgroups); thus, no statistically significant difference was found in any of the subgroups of men (data not shown). However, it has to be highlighted the clear inverse relationship between IGF-I values and DL-PCBs serum levels evidenced in the youngest subgroup of women. As shown in Table 4, younger women with lower BMI (18–46 years and BMI <27 kg/m^2^) who showed undetectable levels of DL-PCB-156 had higher IGF-I serum levels than younger women with lower BMI who showed detectable levels of this contaminant (*p*<0.001). Furthermore, a clear inverse association between IGF-I and ∑DL-PCBs serum values was evident in this age-BMI-subgroup of women; thus, this subtype of women in tertile 1 had higher IGF-I serum levels than those in tertiles 2 and 3 (*p*=0.017); and consequently, ∑TEQs showed a similar inverse relationship. Similar results were observed for TEQs; the lowest ∑TEQs (tertile 1) had higher IGF-I serum levels than those with higher ∑TEQs (tertiles 2 and 3) (*p*=0.019). This inverse association was observed even when PCBs were considered as a continuous variable. Thus, in this subset women, serum IGF-I values decreased with increasing PCB-156, ∑DL-PCBs and ∑TEQs (R=−0.329, *p*<0.005; R=−0.317, *p*=0.008; and R=−0.303 *p*=0.011, respectively).

**Table 4:** Arithmetic means and their 95% confidence intervals (95% CIs) of IGF-I (ng/ml) by serum levels of DL-PCB 156 (ng/g lipid), ∑DL-PCB (ng/g lipid) and ∑TEQs (pg/g lipid) in women, according to age and BMI categories.

		Women (18–46)				Women (>46)			
		N	BMI (<27)IGF-I mean (95%CI)	N	BMI (>27)IGF-I mean (95%CI)	N	BMI (<27)IGF-I mean (95%CI)	N	BMI (>27)IGF-I mean (95%CI)
DL-PCB 156	ND	43	215.2 (199.0–231.3)	11	181.9 (146.6–217.1)	14	168.1 (140.8–195.4)	34	144.3 (126.1–162.5)
	D	24	170.0 (148.4–191.7)	13	166.1 (133.7–198.4)	26	142.5 (122.6–162.5)	37	130.7 (113.3–148.1)
	*p*		0.001		ns		ns		ns
∑DL-PCB	T1	32	219.2 (200.1–238.4)	10	186.1 (148.1–224.1)	8	158.5 (121.1–195.8)	27	136.5 (116.1–156.7)
	T2	21	183.8 (160.1–207.5)	8	164.4 (119.6–209.2)	15	155.3 (127.6–183.0)	18	153.7 (128.8–178.6)
	T3	14	175.7 (146.7–204.8)	6	163.9 (115.4–212.4)	17	144.8 (118.6–170.9)	26	126.6 (105.9–147.3)
	*p*		0.017		ns		ns		ns
∑TEQs	T1	32	219.2 (200.1–238.4)	10	185.0 (147.3–222.7)	8	158.5 (121.1–195.8)	27	136.5 (116.1–156.7)
	T2	21	180.9 (157.2–204.6)	9	172.2 (129.8–214.7)	15	155.3 (127.6–183.0)	19	153.2 (129.0–177.5)
	T3	14	179.9 (150.9–209.0)	5	151.9 (98.2–205.5)	17	144.8 (118.6–170.9)	25	125.8 (104.7–146.9)
	*p*		0.019		ns		ns		ns

Adjusted means of IGF values were obtained by variance component analysis. IGFBP-3 and age or BMI were included in the model as cofounding factors. T1: first tertile of the distribution; T2: second tertile of distribution; T3: third tertile of the distribution; ND: non-detectable; D: detectable; ns: non-significant.

## Discussion

The GH-IGF-I system is affected by environmental changes and is a probable target for endocrine disrupting compounds that may impair many physiological processes. As far as we know, this is the first study to assess the potential effects of DL-PCB exposure on IGF-I, in a general population sample. Our results suggest a modest inverse association between DL-PCBs and IGF-I serum levels, in a subgroup of population formed by young women with low BMI. Nonetheless, due to the fact that this is a cross-sectional study, our results pointing to the possibility that DL-PCBS could exert a negative modulation on GH/IGF system must be taken as an indication of a potential causal relationship.

IGF-I serum levels vary extraordinarily with age [9], [10]. Conversely, a positive correlation between age and serum PCB levels was observed [23], [40], [41]. In this study, we observed an age-dependent inverse association between IGF-I serum levels and DL-PCB 156, ∑DL-PCBs and ∑TEQs, where young women with BMI less than 27 and higher DL-PCBs contamination had lower levels of serum IGF-I.

The IGF-system shows sexual dimorphism [7], although the influence of gender is still not clearly understood [8]. Increases in GH and IGF-1 levels are positively correlated with sex steroids in both sexes during puberty and the influence of these steroids on the GH–IGF axis is believed to be a major determinant in the growth spurt at this time of life [42]. No significant difference between genders in serum IGF was found in our population.

Dioxin-like compounds are considered to be endocrine disrupters [43], [44], [45]. In human male serum, high levels of DL-PCBs were associated with decreased estrogen receptor-mediated activity and increased dioxin-like activity [46]. Our results suggest that the potential negative role of DL-PCBs on the IGF system is influenced by gender and age, with the most evident results in a women-subgroup that assumedly have the highest estrogen levels (women between 18–45 years). The fact that women at their fertile age (18–45 years) are most susceptible to the negative modulation of DL-PCBs on the IGF system is of concern, because of the potential hazard of DL-PCB toxic effects on fetuses at critical stages of development are well documented [47].

The influence of BMI on serum IGF-I levels is well known [6], [11], [12]. In a recent study, BMI values with the highest IGF-I serum levels were reported in men with BMI of 22.5–25 kg/m^2^ and in women with 27.5–30 kg/m^2^ [39]. The Canary Islands population has a rather high proportion of over-weighted and obese people (more than 60%; see Table 1) [48] and subjects with higher BMIs have higher levels of DL-PCBs [23]. Our present results reinforce the importance of BMI on both IGF-I and DL-PCBs serum levels. Thus, only women with high IGF-I levels, i.e. women between 18–45 years old, with BMI below the population mean (27 kg/m^2^) showed an evident inverse association between DL-PCBs and IGF-I serum levels.

There is a crosslink between the IGF-system and other hormonal systems. IGF-I and insulin have complementary roles in the regulation of blood glucose [49]. Interestingly, animal and human cell studies suggest that diverse PCBs and dioxins alter glucose and insulin metabolism. Moreover, PCB 156 and other DL-PCBs have been associated with higher risk of developing type 2 diabetes mellitus [28], [50], [51], [52]. It can be speculated that the negative association between DL-PCBs and the IGF system activity might be related with the low IGF-I serum levels described for diabetic patients. Furthermore, an inverse association between PCB 156 and serum levels of thyroid hormones has been described in one study [53]. The interaction between DL-PCBs, the IGF-system and other hormones could play a potential role in the development of important chronic diseases.

Our results agree with previous studies in the observation that serum IGF-I levels may be modulated by exogenous factors, such as environmental pollutants [14], [38], [54], [55]. These results agree with some previous reports of our research group showing lower IGF-I serum levels in subjects with higher levels of organochlorine pesticides in a way that is highly influenced by gender, age and the involved chemical or combination of chemicals [14], [33]. The relative importance of the effects of pollutants on biological systems is not well understood, especially when complex chemical mixtures are taken into account [56]. Because of such complexity, the mixture of different components with agonistic/antagonistic effects and the limited knowledge about the biological effects of most PCBs may hamper the interpretation of our findings.

Because the liver is the main source of circulating IGF-I, the inverse relationship between PCBs and IGF could be the consequence of a direct effect of these environmental pollutants on the liver [54]. In this context, cytochromes CYP1A1 and CYP2B are activated by DL-PCB congeners, including PCB156, through the binding with the aryl hydrocarbon receptor (AhR) [57].

Nutrition is a major regulator of circulating IGF-I [9]. The presence of environmental pollutants in food has been recently documented in our region [58]. Diet seems to be the major DL-PCBs source for humans [23] with fish and dairy products currently considered to be the major source, according to the European Food Safety Agency in 2010 [59]. Although fish consumption is low in the Canary Islands, the daily intake of dairy products is particularly high [48]. Interestingly, serum IGF-I levels have been found to be highly correlated with the intake of dairy products [60]. Because of this situation, further studies on the influence of dietary habits on the DL-PCBs-IGF relationship are worth the effort.

In summary, we found a significant negative association between serum levels of DL-PCBs and the IGF system in younger women with lower levels of BMI. This result suggests that DL-PCBs, especially DL-PCB-156, could negatively modulate the IGF system in this subgroup of patients. Although some PCBs are known to be hormonally active agents, a plausible mechanism for their effects on the IGF system has not been elucidated yet, a purpose that appears especially challenging when other well-known determining factors – such as gender, age, BMI or diet – are considered. Since both DL-PCBs exposure and the IGF system seem to play a role in some prevalent chronic diseases, studying the effects of DL-PCBs on the IGF system seems to deserve further effort, especially taking into account the implications of this issue for public health decisions. However, the present results concern only a subset of individuals, and other alternative explanation should be taken into account; thus, these results should be interpreted with caution, and the study must be considered as a hypothesis-generating study. Further prospective experiments are needed to confirm those results.
